# Establishing a Robot-Assisted Liver Surgery Program: Early Experience from University Medical Center Ljubljana

**DOI:** 10.3390/medicina62010018

**Published:** 2025-12-22

**Authors:** Miha Petrič, Živa Nardin, Jan Grosek, Aleš Tomažič, Boštjan Plešnik, Blaž Trotovšek

**Affiliations:** 1 Department of Abdominal Surgery, University Medical Center Ljubljana, Zaloska 7, 1000 Ljubljana, Slovenia; jan.grosek@kclj.si (J.G.); ales.tomazic@kclj.si (A.T.); bostjan.plesnik@kclj.si (B.P.); blaz.trotovsek@kclj.si (B.T.); 2 Faculty of Medicine, University of Ljubljana, Korytkova 2, 1000 Ljubljana, Slovenia; ziva.nardin@gmail.com

**Keywords:** robot-assisted, liver surgery, implementation, minimal invasive liver surgery, structured program

## Abstract

*Background and Objectives*: Robot-assisted procedures represent a significant advancement in minimally invasive liver resection techniques. Nonetheless, the introduction of a novel surgical technique in a new environment necessitates meticulous planning and a gradual, stepwise approach. This study describes the adoption of a robotic surgical platform for liver resection at a high-volume tertiary care center. *Materials and Methods*: We retrospectively analyzed data that had been prospectively collected from fifty robot-assisted liver resections. Descriptive statistics, including frequencies, percentages, means/medians, and standard deviations, were employed for description and summary. *Results*: The median operative duration was 166 min (range: 85–400 min), with an average intraoperative blood loss of 200 mL (range: 50–1000 milliliters). Intraoperative or postoperative blood transfusion was required in 8% of patients. Conversion to open resection was necessary in one patient (2%). The mean duration of hospitalization was 5 days (range: 3–20 days), with a 30-day readmission rate of 6% and no mortality within 90 days. Postoperative complications classified as Clavien-Dindo grade 3 or higher were observed in five patients (10%). The mean tumor size varied according to pathology: 58.5 mm (range: 30–120 mm) in the hepatocellular carcinoma group; 27.4 mm (range: 10–32 mm) in the secondary malignancy group; and 42.6 mm (range: 24–60 mm) in the intrahepatic cholangiocarcinoma group. The median number of lymph nodes harvested during lymphadenectomy (IHHCA/GBCA) was 5.4, ranging from 1 to 11. The R0 resection rate for malignant tumors was 88.2% (of 30/34). *Conclusions*: This study validates the safe integration of robot-assisted surgery into liver disease treatment, supported by our initial experience. Despite its technical advantages, robotic-assisted liver surgery remains complex and demanding. Structured robotic training within established programs, meticulous patient selection, and a stepwise implementation approach are critical during the early phases to optimize the outcomes.

## 1. Introduction

In 2008, Gullianoti et al. [1] published a pioneering report on using a robotic platform for liver surgery. A decade later, the same team conducted a series of 70 hepatic resections, with one-third classified as major liver resections [2], and demonstrated that robotic liver surgery yielded outcomes comparable to the open method. Following these initial reports, the robotic approach to liver surgery was validated as both safe and efficient for minor [3] and major [4] liver resections, as well as in complex clinical scenarios such as central liver resections [5,6]. Additionally, it was found to be feasible, safe, and reproducible, with significantly reduced blood loss and shorter hospital stays compared to the open approach for obtaining liver grafts in living-related liver transplantation [7]. In 2018, the first international consensus conference on robotic liver resection (RLR) concluded that, despite certain controversies, such as the cost-effectiveness of RLR, the learning curve associated with RLR, and the outcomes of RLR for challenging liver segments, robotic liver surgery (RLS) is both safe and feasible [8]. Furthermore, it is associated with outcomes comparable to those of open or laparoscopic approaches for both minor and major liver resections for malignant or premalignant diseases [8]. A seminal study by Sucandy et al. [9] demonstrated that robotic-assisted liver surgery is associated with excellent outcomes. Nonetheless, the introduction of a novel surgical technique in a new environment necessitates meticulous planning and a gradual, stepwise approach. It is of utmost importance to recognize that the implementation of a new method may be associated with potential harm to patients, affecting both short- and long-term oncological outcomes and quality of life. This study aimed to evaluate the safety and feasibility of implementing a robotic surgical platform for liver surgery at a high-volume tertiary university hospital in a small European country. The secondary objective was to compare the outcomes with those reported in the literature and contrast them with the results of open liver surgery conducted at our institution.

## 2. Materials and Methods

An analysis was conducted on prospectively collected data from the first 50 consecutive cases of robot-assisted liver resection performed at the Department of Abdominal Surgery, University Medical Center, Ljubljana, from August 2022 to April 2025. Data were gathered on the patients’ general characteristics, including age, sex, body mass index (BMI), etiology of liver disease, and American Society of Anesthesiology (ASA) score [10]. Intraoperative parameters, such as the duration of the surgical procedure, blood loss, conversion, and resected liver segments according to the Couinaud classification [11], were also recorded. Postoperative parameters included length of hospitalization, postoperative complications classified according to the Clavien-Dindo classification [12], 30-day readmission rate, and 90-day mortality rate. Postoperative liver failure [13], bile leakage [14], and hemorrhage [15] were defined using the International Study Group of Liver Surgery (ISGLS) grading system. Additionally, data on post-surgery oncological outcomes were collected, including tumor histology, the number of retrieved and positive lymph nodes when lymphadenectomy was accompanied by liver resection, and the radicality of resection (R0/R1). We classified our patient cohort according to the Brisbane classification [16] of liver resection nomenclature and the IWATE difficulty scoring system [17].

### 2.1. Development of a Robot-Assisted Liver Surgery Program at UMC Ljubljana

MP and BP participated in a formal, structured training program at UT Southwestern in Dallas, TX, USA. All but two robot-assisted liver procedures were performed by MP, with the two left lateral sectionectomies conducted by BP. In most cases, BP served as the bedside assistant. MP is an experienced hepato-pancreato-biliary surgeon, proficient in open liver surgery, and liver transplant surgery, with limited experience in laparoscopic liver surgery, having performed 15 procedures, primarily metastasectomies, segment 6 resections, and left lateral sectionectomies. The first liver resection was performed in August 2022, involving the removal of a small adenoma in a male patient. Following the initial phase of the learning curve, MP visited Iswanto Sucandy, a renowned high-volume robotic liver surgeon at Advent Health Tampa, Tampa, FL, USA, to gain insights and techniques for establishing a successful robotic liver program. In retrospect, this visit was crucial for accelerating the development of the liver surgery program at our institution.

### 2.2. Standard Operative Protocol for Robot-Assisted Liver Surgery

The da Vinci Xi robotic platform (Intuitive Surgical, Sunnyvale, CA, USA) was utilized for robot-assisted hepatic procedures. Our objective was to standardize all surgical steps and procedures through comprehensive training of all surgical team members, including the anesthesiology team and the scrub nurses. We planned and executed all liver resections in accordance with the established surgical and oncological protocols. The inclusion criteria comprised patients older than 18 years who were able to understand and sign the informed consent form. All patients were presented at a multidisciplinary meeting, where the indication for surgical treatment was determined. For the initial 20 cases, we selected patients without prior upper gastrointestinal surgery, those presenting with small malignant tumors and premalignant conditions, and those possessing favorable liver anatomy. After case 20, the only remaining contraindications were the need for trisectomies, previous major upper GI surgery, or prior liver surgery. One day prior to surgery, all patients received a dose of indocyanine green (IGC) to improve intraoperative tumor visualization, unless contraindicated due to iodine allergies. Additionally, if a major liver resection was planned, a dynamic liver function test with an indocyanine clearance test was conducted [18] to assess and avoid resection in patients at risk for postoperative liver remnant failure.

**Figure 1:**
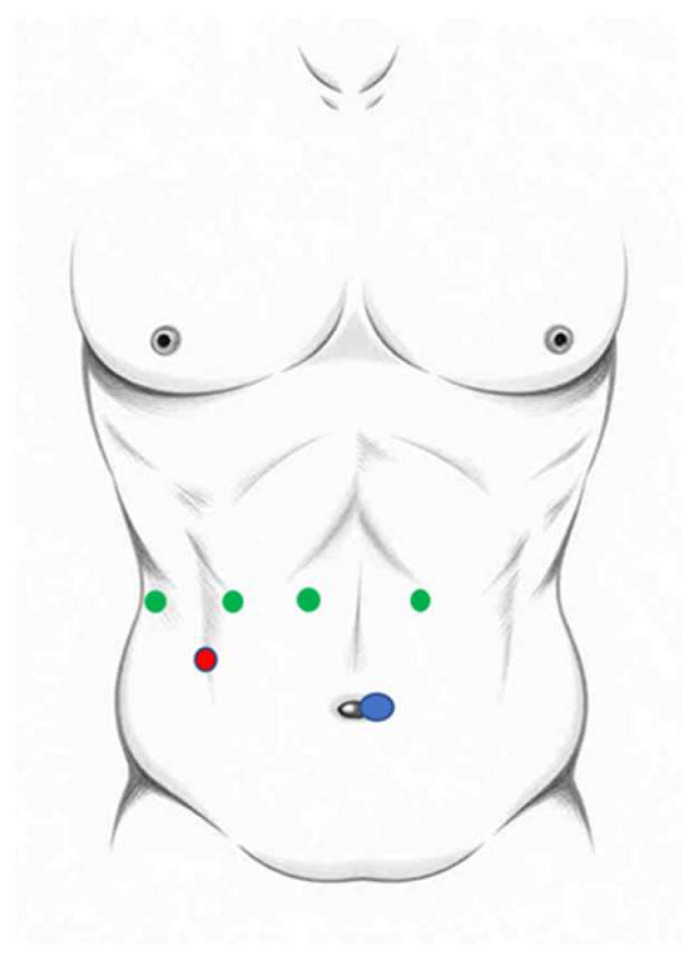
Trocars position for robot-assisted liver resection; 8 mm robotic arm ports (green), Air Seal^®^ port (blue), assistant port (red; 5, 11 or 15 mm).

The patient underwent endotracheal intubation and was secured in the French position, with pressure points added to prevent pressure-induced injuries to the skin and tissues. Initially, central venous catheters were routinely utilized to monitor central venous pressure below 3 mmHg during the first 20 procedures. However, as knowledge and experience were gained, the application of central venous catheters became limited to technically major procedures, significant liver resections involving four or more segments, or when the patient’s condition and comorbidities warranted their use. A urinary catheter was inserted. Prior to robotic docking, the patient was positioned in a 15-degree anti-Trendelenburg position with a 5-degree tilt to the left or right, depending on the planned liver resection. A periumbilical skin incision was made, and pneumoperitoneum was established at 12 mmHg pressure. A 12 mm Air Seal port was inserted. Laparoscopy of the abdominal cavity was performed to rule out distant metastases and signs of non-resectability or extra-hepatic tumor spread. The positions of the four 8 mm robotic arm ports and an assistant port were marked and inserted under direct visualization (Figure 1). The positioning of the robotic camera was adjusted based on the type of liver resection between arms 2 and 3.

In instances where it was necessary, perihepatic adhesiolysis was conducted, and the falciform ligament was dissected. Partial mobilization of the liver attachments was performed to facilitate comprehensive exposure of the liver for ultrasound examination. Intraoperative ultrasound was performed using the integrated Tilepro^TM^ application. Firefly^TM^ immunofluorescence imaging was used to visualize the bile ducts and liver tumors. Ultrasound-guided marking of the tumor and other critical structures was performed to ensure safe and radical liver resection. The lesser omentum was transected, and a Foley catheter, sized 16 or 18, was routinely used for the Pringle maneuver. The Pringle maneuver was employed in the majority of cases (43 out of 50), except when metastasectomy or tumorectomy was performed. In cases where lymphadenectomy was necessitated by oncological considerations (intrahepatic cholangiocarcinoma, gallbladder carcinoma), the procedure was performed from the celiac trunk along the hepatic artery, hepatoduodenal ligament, and retro-pancreatic area. For left-sided intrahepatic cholangiocarcinoma, lymphadenectomy was performed along the left gastric artery. The lymph nodes were placed in a bag and retrieved at the conclusion of the surgical procedure through a mini laparotomy (either Pfannenstiel or median). The subsequent step involved the full mobilization of the liver segment to facilitate safe dissection. In the context of formal hepatectomy procedures, such as right, left, and left lateral sectionectomy, an extrahepatic Glissonean pedicle approach was used. For anatomical liver resections, including segmentectomy and bi-segmentectomy, either an extrahepatic or intrahepatic Glissonean pedicle approach was used. Dissection was consistently performed using the Pringle maneuver. The main branches of the portal vein were ligated using Silk 3-0 in conjunction with Hem-o-lok^®^ clips and subsequently divided. The appropriate arterial branches were clipped with Hem-o-lok^®^ clips and divided. Intraoperative ultrasonography was performed to ensure correct vascular occlusion. Following vascular occlusion, an ICG test was performed to delineate the non-perfused area, and the resection margins were marked. Ultrasound was used again to ensure the radicality of the procedure. Initially, a Cavitron Ultrasonic Surgical Aspirator (CUSA, Integra LifeSciences (Ireland) Limited) was used for parenchymal transection. However, owing to limited resources, specifically the availability of only one CUSA machine and the requirement for a second experienced CUSA operator, we transitioned to the crush–clamp technique utilizing Maryland bipolar forceps and scissors. Smaller structures were divided using a vessel sealer, whereas larger structures were divided using Hem-o-lok^®^ clips or ligature. In cases of parenchymal bleeding, a hemostatic suture was applied using Prolene 3-0 or 4-0. During an anatomical right or left hepatectomy, the Endo GIA (Medtronic) stapler with a vascular load was used to transect the main bile duct, whether left or right. Furthermore, the right and left hepatic veins were severed using Endo GIA (Medtronic) vascular loads. At the conclusion of the liver parenchymal transection, an ICG test was performed to assess potential ischemic areas, and final hemostasis and biliostasis were achieved. The transection surface was sprayed with a hemostatic agent, and, if feasible, the falciform ligament was used as a patch over the hilar structure.

Drains are routinely used along the resection lines. The specimens were placed in an endo bag and retrieved through a Pfannenstiel or mini medial incision. The postoperative protocol was followed in accordance with the established ward guidelines, which emphasized early recovery and mobilization of the patient. Patients were permitted to consume solid food orally on the following day, and the removal of drains and intravenous therapy was conducted as soon as the patients’ clinical conditions permitted. Potential complications were managed according to established principles.

Descriptive statistics such as frequencies, percentages, mean/median, and standard deviations were used for description and summary.

## 3. Results

Over a 32-month period, we performed 50 consecutive robot-assisted liver resections. The baseline demographic and clinical characteristics of the study population are shown in Table 1.

### 3.1. Perioperative Outcomes

In accordance with the Brisbane nomenclature, we performed seven metastasectomies/tumorectomies, 17 segmentectomies, 16 bisegmentectomies, eight left hemihepatectomies (including one with S1 resection), and one right hemihepatectomy. Comprehensive data on perioperative outcomes are presented in Table 2.

According to the IWATE difficulty scoring system16, minimally invasive liver resections are classified into four levels of difficulty: low (L), intermediate (I), advanced (A), and expert (E). In our study, we conducted 7 resections at the low difficulty level, 29 at the intermediate level, 11 at the advanced level, and 3 at the expert level. Table 3 provides a comparison between the different IWATE categories.

In one patient (no. 12), surgery was conducted for a giant hemangioma in the right lobe following preoperative embolization of the feeding artery. Conversion was necessitated due to intraoperative bleeding. The average high-dependency unit stay was 1 d, ranging from 0 to 5 days. The median hospital stay lasted 5 days, ranging from 3 to 20 days. The complication rate was 26% (13/50), with major postoperative complications classified as Clavien-Dindo grade >3, occurring in 10% (5/50) of the cases. Three patients (6%) required percutaneous drainage due to bilomas or abscesses. One patient experienced postoperative bleeding from the assistant trocar site, likely involving the inferior epigastric artery, which required surgical intervention to address. We performed four simultaneous resections in patients with synchronous colorectal malignancies and liver metastases (patients 13, 44, 46, and 47). In two cases (patients 13 and 44), we performed simultaneous robot-assisted right hemicolectomy and liver metastasectomy, specifically a segment 4b metastasectomy in one patient and a left lateral sectionectomy in the other. One patient (patient 46) developed a clinically significant anastomotic leak, which required reoperation using the Hartmann procedure after robotic-assisted resection of the rectosigmoid colon with a left lateral sectionectomy. Another patient (patient 47) with rectal cancer underwent a diverting ileostomy due to obstructing rectal cancer, followed by neoadjuvant systemic treatment and simultaneous robot-assisted low anterior resection with segment 1 resection. Another patient who underwent right hepatectomy for an 11 cm HCC developed grade A postoperative liver failure (Clavien-Dindo grade 2). The 30-day readmission rate was 6%, with three patients requiring readmission. One patient was readmitted due to a recurrent abscess at the liver resection plane, necessitating further percutaneous drainage, while another was readmitted for a suspected infected fluid collection at the resection margin and treated conservatively with antibiotics. No post-operative mortality was observed within 90 days.

In comparison to unpublished hospital data from the most recent 471 liver resections at UMC Ljubljana (2019 to 2024), which reported a median hospital stay of 7 days (range: 1–110), an overall complication rate of 27.6%, a major complication rate of 13.7%, and a 90-day mortality rate of 1.7%, the robotic approach yields outcomes that are not inferior to the established open approach.

### 3.2. Short-Term Oncological Outcomes

In our cohort, 19 patients (38%) underwent surgery for benign or premalignant conditions, whereas 62% underwent surgery for primary or secondary malignancies of the liver. Table 4 presents a detailed etiological classification of liver diseases.

The mean tumor size was 58.5 mm (range: 30–120 mm) in the hepatocellular carcinoma (HCC) group, 27.4 mm (range: 10–32 mm) in the secondary liver malignancy group (colorectal or neuroendocrine neoplasm), and 42.6 mm (range: 24–60 mm) in the intrahepatic cholangiocarcinoma (IHHCA) group. The median number of lymph nodes harvested during lymphadenectomy (IHHCA/GBCA) was 5.4, ranging from 1 to 11. The R0 resection rate of 34 patients treated for malignant tumors was 88.2% (30/34), and four patients exhibited an R1 resection margin. One patient with HCC had a 1 mm resection margin with a thermal injury. Two patients with HCC had a resection margin of less than 1 mm. In one instance of multifocal HCC, four tumors were found in segment 5 and at the junction of segments 4 and 5, where a formal segmentectomy of segment 5 was performed, along with marginal resections of segment 4 (R1 margin). The other case involved an 83-year-old man with HCC in segment 4b and visible liver cirrhosis. One patient with adenocarcinoma of the ascending colon and synchronous liver metastasis had a 0.5 mm margin of liver metastasis; however, she had positive lymph nodes in the hepatoduodenal ligament, with one node encroaching on the left hepatic artery. She is currently undergoing continuous systemic therapy due to systemic disease progression in her lungs. Twenty months post-surgery, the patient with multifocal hepatocellular carcinoma (HCC) exhibited diffuse liver progression and is currently undergoing systemic therapy with Atezolizumab and Bevacizumab. In contrast, the other two patients with HCC have shown no evidence of tumor recurrence. As of December 2025, all patients included in the study are still alive, with follow-up periods ranging from 8 to 40 months.

## 4. Discussion

Our study demonstrates that with meticulous planning and a step-by-step approach, a robotic surgical platform can be effectively implemented in minimally invasive liver surgery. Robot-assisted liver surgery can be performed safely and efficiently, yielding outcomes comparable to those of the open and laparoscopic approaches. Every novel surgical approach should be carefully adopted through a strictly controlled process to ensure that the potential learning curve does not interfere with treatment outcomes, which should be comparable to those established in open and laparoscopic surgery. To achieve this objective, the implementation of any new technique should adhere to the framework proposed by Søreide et al. [19], which formulates the concept of survival in surgery. This framework is based on four essential pillars: surgical science, education, implementation, and nonsurgical skills [19].

A seminal study by Sucandy et al. [9] revealed that robotic-assisted liver surgery yields excellent outcomes. The study involved 530 robotic hepatectomies, with over half categorized as major or technically major resections. Postoperative results showed a low overall morbidity rate of 8%, with major complications (Clavien-Dindo score > III) at just 4% [9]. The average hospital stay was 4.0 ± 3.7 days, and the 30-day readmission rate stood at 86 (16%) [9]. Among patients undergoing liver resection for malignant tumors, R0 resection was achieved in 95% of cases [9]. The overall survival rates for colorectal liver metastases were 82%, 65%, and 59%; for hepatocellular carcinoma, they were 84%, 68%, and 60%; and for intrahepatic cholangiocarcinoma, they were 79%, 61%, and 50% at 1, 3, and 5 years, respectively [9]. A meta-analysis and systematic review aimed to assess the effectiveness of the robotic approach for patients with hepatocellular carcinoma (HCC) compared to open or laparoscopic methods [20]. The results showed that robotic liver resection (RLR) was on par with open liver surgery (OLS) and laparoscopic liver surgery (LLS) regarding overall complication rates and complications, such as liver failure or bile leak [20]. A meta-analysis involving 1093 patients, comprising 345 patients who underwent robotic liver resection (RLR) and 748 patients who underwent laparoscopic liver resection (LRL) for malignant liver etiology, revealed that RLR was associated with longer operative durations, larger tumor sizes, and reduced conversion rates [21]. No significant differences were observed in terms of estimated blood loss (EBL), blood transfusion rates, length of hospital stay, R0 resection rates, or complication rates [21]. The authors concluded that the robotic approach is both safe and feasible compared to the laparoscopic approach [21]. In 2018, the International Consensus Conference on robotic liver resection (RLR) concluded that, despite certain controversies, such as the cost-effectiveness of RLR, the learning curve associated with it, and the outcomes for complex liver segments, robotic liver surgery (RLS) is both safe and feasible [8]. Furthermore, it yields outcomes comparable to those of open or laparoscopic approaches for both minor and major liver resections in cases of malignant or premalignant diseases [8]. The 2024 IEGUMILS meeting in Brescia concluded that RLR is both safe and efficient for selected patients with colorectal liver metastases, hepatocellular carcinoma (HCC), intrahepatic cholangiocarcinoma, and benign liver tumors, irrespective of the type of liver resection (minor, major, or major technical), when performed by experienced surgeons [22].

The successful implementation of a robotic surgical platform in liver surgery, achieving outcomes comparable to those of open surgery, depends on education, structured training, and a methodical approach to the procedure. The initial phase involves becoming familiar with the robotic platform, which can be effectively achieved through virtual reality training. Raison et al. [23] demonstrated that a structured program of procedural VR simulations is effective and correlates with the successful transfer of technical skills to real-life situations. Individuals with prior surgical experience acquire kinematic properties more rapidly than novice surgeons [24]. The literature suggests that surgeons and surgical units experienced in open or laparoscopic approaches can facilitate the implementation of robotic surgical platforms, resulting in a lower conversion rate and a steeper learning curve [25]. The robotic platform allows for a relatively brief learning curve for procedures of low to intermediate complexity, regardless of prior laparoscopic experience, although such experience is still considered essential [26]. Establishing an effective learning curve for robotic liver surgery is challenging because of the diverse range of potential procedures, including formal hepatectomies, segmentectomies (encompassing both minor and technical major), subsegmentectomies, and vascular and biliary resections. Consequently, several established scoring systems facilitate comparisons between different liver procedures. Among the most frequently utilized are the Southampton [27] and IWATE scoring systems [17], both initially developed for laparoscopic liver resections. In 2024, a unique robotic-specific difficulty scoring system, the Tampa Difficulty Score [28], was developed by a group in Tampa. Their single-center experience outlined a progression in skill acquisition and case complexity, categorized as competency (cases 1–20), proficiency (cases 21–30), early mastery (cases 31–65), and full mastery (cases 66–100) [29]. Sucandy et al. [30] suggested that the learning curve for minor resections was 75 cases, for major resections was 100 cases, and for technically challenging minor resections was 57 cases. Another influencing factor is the type of robotic liver parenchyma resection. The most employed techniques are the crush clamp technique, CUSA, and SynchroSeal methods [31]. No techniques have proven to be superior to the others, and they can be combined to achieve optimal outcomes [32]. The impact of hospital volume on liver surgery outcomes remains debatable. Some studies have reported no effect of hospital volume on postoperative outcomes [33], while others have suggested that the number of liver surgeries performed at a hospital significantly influences patient outcomes [34]. Factors affecting these outcomes include a deeper understanding of anatomy, appropriate patient selection, advancements in surgical techniques, perioperative management, and multimodal management of postoperative complications [35]. The successful establishment of a robotic surgery program depends not only on the lead surgeon’s expertise but also on the creation of a dedicated team, including assistants, anesthesiologists, scrub nurses, and ward staff [36]. It is essential to provide comprehensive training and education for all team members, develop detailed operational manuals for operating room teams, and establish protocols for postoperative recovery and credentialing [36]. Additionally, fostering an environment that encourages the exploration of potential improvements is crucial for ensuring safe and effective clinical practice [37].

In August 2022, our institution performed its first robotic liver resection, excising a small adenoma located in segment 3 of a male patient. Our goal was to adopt a systematic, stepwise approach to establish a learning curve. However, limited access to robotic platforms and the simultaneous operation of laparoscopic and open liver programs pose challenges in securing a sufficient number of straightforward cases. In the second case, a 12 cm hepatocellular carcinoma (HCC) was identified in the right lobe of the liver. We recognize that this procedure is inherently complex and should be performed only after establishing a learning curve. Nonetheless, the primary aim of this initial procedure was to mobilize the right liver, perform surgical exploration of the hepatoduodenal ligament, and manage extrahepatic inflow if feasible. Owing to our extensive experience in open surgery and proficiency in laparoscopic techniques, we encountered no intraoperative complications, thereby enabling the successful completion of the resections. The authors acknowledge that an appropriate learning curve should begin with simple, minor liver resections and gradually progress to more complex procedures. Despite not fully aligning with the suggested learning path and implementation, we continue to achieve results that meet the established published benchmarks. A recent study by Goh et al. [38] which analyzed 1654 RLR-defined benchmarks, reported the following metrics for robotic left lateral sectionectomy (RLLS), left hepatectomy (RLH), right hepatectomy (RRH), and right posterior sectionectomy/H67 (RRPS): operation time (190, 323, 474, 413 min), open conversion rate (0.0, 0.0, 1.3, 0.0)%, estimated blood loss (100, 250, 600, 550 mL), blood transfusion rate (0.0, 0.0, 20.0, 29.2)%, postoperative major morbidity (0.0, 0.0, 20.9, 16.7)%, 90-day mortality (0.0, 0.0, 0.0, 0.0)%, and textbook outcome (12.5, 24.3, 0, 0)%. In our initial series, RLLS (eight cases) showed an operation time of 112 min, 91 mL blood loss, a 14.3% transfusion rate, 14.3% major morbidity, and no mortality. The RLH (6 cases) had an operation time of 201 min, 107 mL blood loss, no transfusion, 16% major morbidity, and no mortality. The RRH case lasted 342 min with a blood loss of 300 mL, morbidity classified as Clavien-Dindo 2, and no mortality. Evidence from the Netherlands indicates a significant increase in the adoption of robotic liver surgery, with its prevalence rising from 0.2% in 2014 to 11.9% in 2020 [39]. The nationwide outcomes are comparable to those of other surgical techniques, with a median blood loss of 150 mL (interquartile range 50–350 mL) and a conversion rate of 6.3% (n = 25) [39]. Major complications, classified as Clavien-Dindo grade ≥ III, were observed in 7.0% of cases, and the median hospital stay was 4 days (interquartile range 2–5) [39]. The 30-day/in-hospital mortality rate was 0.8%, and the R0 resection rate was 83.2% [39]. In our cohort, the results align with these reported figures. Notably, the conversion rate was 2%, and major postoperative complications (Clavien-Dindo grade ≥ III) occurred in 10% (5/50) of patients. There were no mortalities in our patient group. In cases with malignant etiology (34/50), an R0 resection was achieved in 30/34 (88.2%) of instances.

Our study has several limitations. The primary concern was the potential bias in patient selection. Specifically, candidates for robotic liver resection were carefully chosen, excluding those with previous major abdominal surgery, chronic liver disease, major vessel infiltration, or a high body mass index (BMI). After 20 cases, the exclusion criteria were previous hepatic surgery and cases requiring trisectionectomies. Nevertheless, this selection process resulted in a successful initial series with outcomes comparable to those of open surgery. The second limitation was the retrospective nature of our study and the relatively low case load.

## 5. Conclusions

In conclusion, we demonstrated the safe implementation of a robotic surgical platform in the field of liver surgery. Although the optimal positioning of robotic modalities in liver disease management is yet to be fully established, our initial experience indicates both the safety and feasibility of the robotic surgical approach for patients with various liver conditions. Patient selection is crucial, particularly during the early phases of developing a robotic program. As a tertiary university center, we are now able to utilize three different approaches—open, laparoscopic, and robotic modalities—to ensure the best possible outcomes for our patients.

## Figures and Tables

**Table 1:** Baseline demographic and clinical characteristics of the study population.

	RLR = 50
Gender (male)	26 (52%)
Age (years, median, range)	65.5 (24–84)
BMI (kg/m^2^, median, range)	27.8 (19.5–40.6)
ASA score	
1	4 (8%)
2	16 (32%)
3	30 (60%)
Liver function	
normal	48 (96%)
Child–Pough A	2 (4%)

RLR—robotic liver resection; BMI—body mass index; ASA—American Society of Anesthesiology.

**Table 2:** Perioperative outcomes.

	RLR = 50
Operative time (min, range)	166 (85–400)
Blood loss (mL, range)	200 (50–1000)
Conversion (n, %)	1 (2%)
HDU (day, median, range)	1 (1–5)
LOH (day, median, range)	5 (3–20)
Transfusion rate (n, %)	4 (8%)
Overall complication (n, %)	14 (28%)
C-D > 3 complication (n, %)	5 (10%)
30-day rehospitalization (n, %)	3 (6%)
90-day mortality (n, %)	0

n—number of patients; HDU—high dependency unit; LOH—length of hospitalization; C-D > 3—Clavien-Dindo grade complications more than 3.

**Table 3:** Robotic liver resection according to the IWATE difficulty scoring system.

IWATE Score System	NoP	OP Time	LOH	Overall CR	C-D > 3
L	7	156	3	1/7 (14.3%)	1/7 (14.3%)
I	29	155	5	7/29 (24.1%)	2/29 (6.9%)
A	11	171	5.5	2/11 (18.2%)	1/11 (9.1%)
E	3	324	13	2/3 (66.7%)	0

L—low; I—intermediate; A—advanced; E—expert; NoP—number of patients; OP—median operative time; LOH—median length of hospital stays; CR—complication rate; C-D > 3—Clavien-Dindo grade > 3.

**Table 4:** Pathology report on disease etiology.

Etiology	No. of Patients	%
Adenoma/BIN	4	8%
Mb. Caroli/hepatolithiasis	7	14%
Hepatic Echinococcosis	3	6%
Hemangioma/cyst	5	10%
Hepatocellular carcinoma	11	22%
IHHCA/GBCA	6	12%
Secondary meta (CRC, NEN)	13	26%
Sarcomatous carcinoma	1	2%

BIN—biliary intraepithelial neoplasia; IHHCA—intrahepatic cholangiocarcinoma; GBCA—gallbladder adenocarcinoma; CRC—colorectal adenocarcinoma; NEN—neuroendocrine neoplasm.

## Data Availability

Data is available on demand.
